# Targeting the Progression of Parkinson’s Disease

**DOI:** 10.2174/157015909787602814

**Published:** 2009-03

**Authors:** J.L George, S Mok, D Moses, S Wilkins, A.I Bush, R.A Cherny, D.I Finkelstein

**Affiliations:** 1 The Mental Health Research Institute of Victoria , 155 Oak Street, Parkville, Victoria 3052; Australia; 2 St Vincent’s Hospital, Melbourne, Fitzroy, Victoria 3065 Australia

**Keywords:** Parkinson’s disease, pathology, redox chemistry, metallo- chemistry, review, iron.

## Abstract

By the time a patient first presents with symptoms of Parkinson’s disease at the clinic, a significant proportion (50-70%) of the cells in the substantia nigra (SN) has already been destroyed. This degeneration progresses until, within a few years, most of the cells have died. Except for rare cases of familial PD, the initial trigger for cell loss is unknown. However, we do have some clues as to why the damage, once initiated, progresses unabated. It would represent a major advance in therapy to arrest cell loss at the stage when the patient first presents at the clinic. Current therapies for Parkinson’s disease focus on relieving the motor symptoms of the disease, these unfortunately lose their effectiveness as the neurodegeneration and symptoms progress. Many experimental approaches are currently being investigated attempting to alter the progression of the disease. These range from replacement of the lost neurons to neuroprotective therapies; each of these will be briefly discussed in this review. The main thrust of this review is to explore the interactions between dopamine, alpha synuclein and redox-active metals. There is abundant evidence suggesting that destruction of SN cells occurs as a result of a self-propagating series of reactions involving dopamine, alpha synuclein and redox-active metals. A potent reducing agent, the neurotransmitter dopamine has a central role in this scheme, acting through redox metallo-chemistry to catalyze the formation of toxic oligomers of alpha-synuclein and neurotoxic metabolites including 6-hydroxydopamine. It has been hypothesized that these feed the cycle of neurodegeneration by generating further oxidative stress. The goal of dissecting and understanding the observed pathological changes is to identify therapeutic targets to mitigate the progression of this debilitating disease.

## 1 INTRODUCTION

Classified as the second most common neurodegenerative disorder, Parkinson’s disease (PD) is a debilitating motor related disease and is presently incurable. Resulting from a gradual progressive degeneration of nigrostriatal neurons, PD affects approximately 1% of the population over 50 years of age [333].

The pathological characteristics of PD require careful differential diagnosis of specific features which are only confirmed *post mortem*. Determined by microscopic examination, the key pathological characteristics of PD are loss of nigral neurons (and loss of pigmentation in this area), and the presence of insoluble proteinaceous cytoplasmic inclusions termed Lewy bodies (LB) and Lewy neurites in the remaining cells. The inclusions are rich in the fibrils of the presynaptic protein alpha synuclein (α-synuclein) and ubiquitin which are thought to arise from the failure of normal degradation in the proteasome pathway (see section 2.3) [197, 280, 381]. Motor symptoms that are associated with the disease are most likely to be caused by a loss of dopamine (DA) producing cells within the substantia nigra pars compacta (SN), in particular, in the interconnections between the SN and the caudate and putamen. The five nuclei which comprise the basal ganglion are; Substantia Nigra (SN), Globus Pallidus (GP), Subthalamic Nucleus (STN), Caudate Nucleus (CN), and Putamen (CPu). The CPu seems to be the most affected structure, losing up to 95% of dopaminergic inputs compared to 80% in the caudate nucleus [184, 214]. Animal models that have shown dynamic rearrangement of the dopaminergic terminals after lesioning suggests that this is likely to be occurring in the preclinical stages of the human disease [114].

In recent times, a new concept in the progression of PD has emerged [45] proposing that a number of nuclei are involved before the SN. The first appearance of disease related symptoms correlates with functional deficits in the lower brainstem and olfactory bulb, then dysfunction progresses up the brainstem to produce classical PD [45]. Changes in other nuclei are observed and are thought to be secondary to the primary disease [175, 176]. The disease progresses until cortical and cognitive changes develop. In this review we will focus on interventions which may prevent the chronic degeneration of the dopaminergic neurons of the SN.

Clinical behavioral symptoms of PD are linked to uncontrolled motor deficits such as akinesia (absence of movement or temporary paralysis), bradykinesia (abnormal slowness of movement), abnormalities in gait, resting tremor and rigidity. Cognitive dysfunction such as speech, executive function and memory loss can also develop as later symptoms.

The study by Braak and colleagues (2003) [45] has also shown that patients that exhibit early stage dysfunction (such as REM sleep disturbance, restless leg syndrome) go on to develop PD within 20 years. In the future, these clinical developments may identify patients that would be suitable candidates for neuroprotective therapies before the SN cells are lost.

PD is a progressive neurodegenerative disease with up to 10% of the remaining dopaminergic cells dying each year [295]. It has been suggested that this progression of the disease explains the loss of responsiveness to drug therapies such as L-dihydroxyphenylalanine (L-DOPA), ultimately failing as the number of DA producing cells falls below a critical level [111]. Most patients benefit from L-DOPA without complications for only approximately five years from the commencement of treatment. Subsequently, the side effects of the treatment become apparent with the percentage of motor fluctuations and dyskinesia ranging from 10% of patients at 5 years, up to 80–90% in later years [265, 266]. Some researchers within the field suggest that pharmacological intervention with neuroprotective therapies in the preclinical stage of PD will have a better chance of prolonging near normal function in patients [200, 264]. New diagnostic tools are currently being developed with several able to provide diagnosis at an earlier stage than is commonly achieved at present [380, 431]. With our expanded clinical perspective on parkinsonism, there is a real chance of utilizing new neuroprotective therapies when they become available. Current studies have hypothesized an interaction of multi-factorial risk factors which can cause the disease. These can be classified as: genetic, environmental, metabolic (oxidative stress; reactive species (RS) production) and biochemical (protein aggregation). New diagnostic techniques and promising neuroprotective pharmacological agents are becoming a reality enabling the next stage in PD therapy, slowing down the progression of SN cell loss in the disease. This review focuses on the possible contributing factors that cause PD, in order to discuss possible strategies to intervene and to slow the progression of PD.

## 2 PUTATIVE CAUSES AND RISK FACTORS INVOLVED IN PD

Various contributing factors such as drugs of abuse, industrial/ agricultural chemicals and genetic mutations have all been known to cause PD or PD type symptoms in humans. Therefore animal models that utilize these factors are valuable  tools to help identify the molecular mechanisms of neurodegeneration that are associated with PD. The exact mechanistic trigger that causes loss of nigral cells found in sporadic PD remains unknown. Many studies have revealed that in combination with a genetic predisposition, contributing factors for oxidative stress (due to the dysregulation in metabolic homeostasis) are involved with the degenerative processes. Although only a minor proportion of these PD patients (<10%) harbor all known genetic deficits, investigation of these mutations may reveal the mechanisms through which a therapeutic target could be identified [400]. With this caveat in mind, the genetic mutations point toward the direction of possible pharmacological targets.

### 2.1 Genetic Factors

PD is primarily a sporadic disorder that is believed to be ‘multi-factorial’ occurring through the involvement of interactions between genetic and environmental factors. Epidemiological studies have shown that while sporadic PD occurs at a later onset of 60 years of age, familial onset tends to develop at a younger age (<50 years), and occurs in approximately 1% of all PD cases [333]. Thus, genetic mutations alone cannot explain the majority of disease cases. However, mutations in specific genes in familial PD have given emphasis to particular genes involved in the molecular pathogenesis of parkinsonism. Six gene loci have been identified that have a PD pathological phenotype (see Table **1**). Mutations in the α-synuclein (α-syn), *Parkin*, PTEN- Induced putative Kinase 1 (*PINK1*), *DJ-1*, Leucine rich repeat kinase 2 (*LRRK2*), ATP13A2 and *UCH-L1* genes have all been shown to be involved in familial PD.

#### α-synuclein

Specifically enriched in the adult brain, the α-synuclein protein is ubiquitously expressed within the central nervous system (CNS) in neurons and glia and is localized in neuronal structures [198]. Belonging to a structurally homologous protein family which includes β- and γ-synuclein, its function has not been well defined. Recent publications have implicated α-synuclein as a neurotransmitter modulator regulating vesicle handling (recycling and docking) [121, 122, 228], synaptic transmission and re-uptake [249]. Structural assays have determined that α-synuclein has the potential to bind to phospholipids particularly on synaptic vesicles [80, 300]. The binding to phospholipids occurs through the N-amphiphatic domain which is a highly conserved region. α-Synuclein knockout animals have deficits in expression of synaptic-associated proteins as well as a reduction in synaptic vesicles [50], but surprisingly only subtle behavioral deficits. Most recently, other novel functions have shown α-synuclein as a protein trafficking chaperone as defects in the gene obstructs ER-Golgi vesicular trafficking [74]. This evidence suggests that α-synuclein plays an essential role in different cellular functions which may be disrupted in PD and other diseases where α-synuclein aggregates are present such as Dementia with Lewy Bodies (DLB) and Multiple System Atrophy (MSA) - (synucleinopathies). The parkinsonian disease phenotype to genotype correlation was firstly linked to a mutation in the α-synuclein gene derived from an Italian kindred [333, 334]. The penetrance in this family alone was 85%, comprising of a single point mutation, a G to A conversion at position 209 which ultimately changed the amino acid code from an Alanine to Threonine at residue 53 (A53T). Since then, two other point mutations (A30P and E46K) have been characterized and shown to segregate with the disease. Whole gene duplication [58, 195] and triplication resulting in over-expression of wild type α-synuclein [368] have been found to cause familial PD, all increase the rate of onset and rate of disease progression [301]. The A53T mutation increases the mean rate of onset of PD (to approximately 46 years of age), muscle rigidity and bradykinesia being the prominent clinical features [14]. Systematic mutational screening of the α-synuclein gene revealed another mutation, A30P, which displayed similar features to those observed in sporadic cases, with classic fundamental behavioural deficits associated with L-DOPA responsive PD [227]. Unlike the other missense mutations identified in this gene, the E46K mutation is linked to LB with dementia [440] which is associated with amyloid fibrilization [156]. The mutations in α-synuclein (A53T, A30P) can form filaments at an increased rate *in vitro* [72]. Wild type α-synuclein undergoes oligomerization when DA is present (in a dose dependent manner). These oligomers are found to be soluble, but are not amyloidogenic [53]. In the presence of iron and copper, wild type α-synuclein can rapidly form soluble SDS resistant oligomers but the formation of amyloidogenic α-synuclein aggregates can be completely inhibited by the addition of DA [53]. This strongly suggests that DA acts as a dominant modulator of α-synuclein aggregation [53]. The structural morphology of the aggregates of α-synuclein differs across genetic mutation species and when copper or iron is present [28]. Interestingly, the three mutations have been shown to reside in potential metal binding sites (for both copper and iron) which could alter the binding of metal-protein complexes, this may be important in fibril formation [29]. In its native state, α-synuclein is an unfolded protein [143, 409, 423] due to an overall low net hydrophobicity [408]. However, like many amyloidogenic proteins such as the beta amyloid protein (Aβ), the α-synuclein protein has a propensity to aggregate specifically *in vitro* to form higher order soluble oligomers, an intermediate species, which in turn further polymerizes into fibrils. Insoluble filamentous aggregates of α-synuclein are the major component of LB in PD and other neurodegenerative synucleinopathies [381]. There is no clear evidence that demonstrates if the development of LB occurs as the cause or a result of the disease and it remains to be established whether the toxic form of α-synuclein is a soluble oligomer, as has been postulated for Aβ in Alzheimer’s disease, or the classic insoluble fibrils. The oligomeric species is considered to be the most neurotoxic, possibly by causing vesicle permeability [416]. On the other hand the accelerated pathology of the α-synuclein mutants (particularly A53T), is associated with an increased rate of self-fibrilization [301].

#### Parkin

Parkin (PARK2) mutants have been linked to autosomal recessive juvenile parkinsonism. An array of mutations including deletions [170, 215, 256], multiplications, rearrangements, missense and several point mutations have now been reported. Parkin mutants present as a wide range of clinical PD phenotypes but differ from idiopathic PD as they tend to be more early onset, with slow progression and neuronal death in the absence of LB formation (see Table **1**) [361]. Shimura and colleagues (2000) [361] showed that Parkin is an E3 ubiquitin ligase the loss of functional activity of which leads to protein accumulation. E3 proteins attach polyubiquitin chains to target proteins that are to be degraded by the proteasome. Mutations that have been described result in greatly diminished Parkin production which in turn results in the failure of Parkin associated substrates to be degraded. However, this fact seems to be confounded as the survival of DA neurons in Parkin knockout mice remains unaffected [145].

#### PINK1

PINK1 was first identified in cancer expression profiles [407] and shown to be localized to mitochondria by both immunofluorescence and western blot analysis [411]. In a study of three PD kindreds, two families harbored a mutation within the kinase domain of the PINK1 gene; a missense mutation and a nonsense mutation. To date, around 40 mutations have been characterized (see Table **1**), most of which have not yet been correlated to a neuropathological phenotype in human PD cases. Many mutations have been discovered in late onset cases, equivalent to sporadic PD cases [4] and also to atypical early onset PD. Mutations found in PINK1 in early onset cases maybe the second most common mutation after Parkin to induce parkinsonism [168]. However, pathogenic PINK1 mutants seem to be a rare cause of early onset PD [344]. The potential PINK1 substrates seem to phosphorylate mitochondrial proteins that may act to preserve mitochondrial integrity [411]. However, these target substrates remain unknown. Expression of mutant forms of PINK1 are said to be loss of function mutations, which suggests that the potential kinase activity of PINK1 is required and therefore highlights the relevance of mitochondrial dysfunction as one of the processes that mediate PD.

Expression of mutations in this mitochondrial kinase, demonstrates a heterogeneous spectrum of pathological phenotypes ranging from early onset to late onset PD. Less than 10% of PINK1 protein has been shown to be found in LB which could be due to poor solubility of the protein and a greater propensity for its aggregation in culture [23]. There have been no *post mortem* studies looking at the neuropathology of PINK1 mutation carriers.

#### LRRK2

Mutations in the LRRK2 gene have been identified in both the sporadic and familial forms of PD and display an autosomal dominant inheritance pattern of PD [452]. More than 30 mutations in the LRRK2 gene have now been reported and many of these reside within a highly conserved functional domain of the protein [85, 86, 452]. It has been determined that LRRK2 possesses kinase activity but little is known about its phosphorylation substrates and its specific role in PD pathogenesis. Expression of LRRK2 is shown in all tissues and at low levels in the brain [316]. The average age of onset in familial cases is the late 50s and 60s, indicating that of all the genes identified in familial PD cases, this gene bears the closest resemblance to sporadic forms of the disease. Varying pathological phenotypes have been observed in PD patients with mutations in LRRK2 and may reflect multiple roles or target substrates for this protein.

#### DJ-1

DJ-1 mutants were first reported in a consanguineous Dutch family [413]. The first mutation identified in this family was a homozygous exon 1 to 5 deletion [413] which effectively caused a deletion of the entire protein. Another reported pathogenic homozygous mutation L166P showed co-segregation with PD in an Italian family [44]. Other reports of DJ-1 mutants (see Table **1**) have been described. The loss of function from specific mutations has been shown to cause autosomal recessive early onset PD. Mutations in DJ-1 are extremely rare occurring in approximately 1-2% of early onset familial cases [3]. There have so far been no reports of any DJ-1 mutations in late onset sporadic cases [3]. DJ-1 is suggested to be co-localized in the mitochondria [446], and is more predominantly up-regulated under high oxidative stress conditions [38]. DJ-1 related cellular processes include, attenuating oxidation [196, 286, 288], RNA binding [181], cell transformation, and androgen receptor signaling [331]. The role of DJ-1 in neuroprotection against the oxidative stress response is of particular interest to PD. Drosophila models which lack DJ-1 show increased susceptibility to oxidative stress and environmental toxins such as paraquat and rotenone [235, 320]. It has been shown that loss of DJ-1 alone cannot sufficiently induce parkinsonism but increases the susceptibility of DA neurons under an oxidative environment [213].

#### UCH-L1

The only mutation described in the UCH-L1 gene was the I93M mutation found in a German family [240]. One other reported polymorphism (S18Y) was found in exon 3 of UCH-L1 [247] but is suggested to be non-pathogenic since it is found in about 20% of the Caucasian population [247]. UCH-L1 mutations have yet to be correlated to a cellular morphological phenotype in PD. However, *in vivo* transgenic mice models with this mutation display gracile axonal dystrophy. This autosomal disease results in axonal degeneration [389], Aβ protein and accumulation of ubiquitinated proteins within the sensory and motor systems [347, 389].

UCH-L1 has a high sequence homology to the gene family that hydrolyzes ubiquitin c-terminals to form monomeric ubiquitin (ubiquitin hydrolase) [241]. It has been suggested that UCH-L1 may have multi-functional capabilities including the recycling of ubiquitin by hydrolyzing ubiquitinated proteins and ubiquitin ligase activity [250].

### 2.2 Environmental Toxins

While the genetic contribution to the disease seems to be only a small proportion of cases, environmental factors are thought to play a pivotal role in PD etiology. In the early 1980’s it was suspected that exogenous neurotoxins caused PD-like symptoms. These gave rise to correlative studies which suggest that long term exposure to common pesticides cause PD-like symptoms. Mitochondrial dysfunction plays a vital role in DA neuron degeneration. Mitochondrial inefficiency is considered to be a result of oxidative stress and is proposed as a primary mechanism for nigral loss following exposure to a range of substances [399]. These neurotoxic agents potentially elicit their effects on mitochondria.

Large scale cohort studies of both chronic and low dose exposures to pesticides have shown a 70% increase in risk of developing PD compared to people not exposed to these chemicals [13]. As both farmers and non-farmers seem to share the same level of risks associated with PD and pesticide exposure, Ascherio and colleagues (2006) [13] suggests that non-farmers could have been exposed to harmful pesticides while gardening. It is becoming apparent that common chemicals, such as Paraquat and Rotenone, could be implicated in some of the PD cases.

#### 2.2.1 Agricultural Toxins

Paraquat (PQ; N,N'-Dimethyl-4,4'-bipyridinium dichloride) is a potential neurotoxin as it has the ability to cross the blood brain barrier [75, 427]. PQ is thought to participate in redox cycling as it can be easily reduced to form free radicals, resembling a structural analogue of a known neurotoxic metabolite 1-methyl-4-phenylpyridinium ion (MPP^+^). Like MPP^+^, PQ selectively destroys dopaminergic neurons. Animal models have shown that systemic [276] and long term infusion of PQ has a toxic effect on rodent dopaminergic neurons [312]. PQ causes a loss of TH positive neurons together with loss of striatal terminal projections, decreases in Dopamine transporter (DAT) and accelerated fibrilization of α-synuclein [260, 410].

The insecticide *Rotenone* is widely used as an *in vivo* animal model of inducing PD. It is thought that the rotenone model is of key importance as the specific neuropathology reflects the selective loss of DA neurons in the SN and LB formation [27, 287, 399]. However the specificity of the model has been called into question as neurodegeneration has also been observed in non-dopaminergic systems [182]. Rats chronically infused with rotenone show severe behavioral deficits characteristic of PD including an unsteady gait and bradykinesia [7, 8]. Chronic low doses of rotenone also show an increase in oxidative stress markers as well as α-synuclein positive LB [360]. Similar behavioral observations have been shown in mice treated with rotenone which do not exhibit LB inclusions or other pathological changes [342].

Rotenone induces the cellular death cascade *via* inhibition of the mitochondrial metallo-protein complex I [27, 359, 360] resulting in oxidative phosphorylation dysfunction. Rotenone causes the inhibition of complex I throughout the brain, however not all investigations have shown loss of SN neurons [27]. The presence of oxidative metabolites in samples from PD tissue is similar to those oxidative metabolites produced by rotenone suggests that oxidative stress could play an important role in the chain of events leading to PD [399].

#### 2.2.2 Drugs of Abuse and Parkinsonism

Drug-induced parkinsonism has been reported in both humans and in animal models [116]. The commonly abused amphetamine derivatives have been reported to damage catecholamine neurons in both humans and in animal models of the disease [193]. Abuse of drugs such as amphetamine, ecstasy and cocaine are known to induce alterations in striatal DA [220] with specific effects on Tyrosine Hydroxylase (TH) positive cells [220, 377, 403] in mice models. Drugs of these sorts have specific modes of action which make them potential catalysts for PD: i) amphetamine inhibits DA uptake causing a marked elevation in extracellular DA. ii) A single dose of cocaine results in elevated levels of hydrogen peroxide and lipid peroxidation products for up to 50 hours [96]. iii) There is increased DA turnover as the result of DA and its metabolites causing an elevation in extracellular DA [319]. iv) The increase in DA production due to amphetamine generates a toxic cascade of free radicals [77]. v) *Post mortem* brain tissue from human chronic cocaine abusers exhibit over-expression of α-synuclein mRNA with a three fold increase in the α-synuclein protein levels in dopaminergic neurons [269] although no data are available on the histopathology of abusers [93]. vi) Amphetamine and similar drugs have been shown to redistribute DA into the cytoplasm where it can be oxidized into semi-quinones and other oxidative metabolites [117]. vii) Drug induced toxicity has been shown to form inclusions containing α-synuclein specifically within the SN [116]. While the use of recreational drugs has raised the possibility of an associated increased risk of developing PD, this link has not been clearly established using population based studies.

#### 2.2.3 MPTP

In the early 1980’s, drug addicts who tried to manufacture their own drugs, instead synthesized MPTP and developed a syndrome symptomatically very similar to sporadic PD (L-DOPA responsive, progressive, with neuronal inclusions) [233, 234]. This neurotoxin has subsequently been extensively used in non-human primates to replicate the cardinal symptoms of PD [354]. Although rodents are more resistant to MPTP than primates, they have contributed greatly to the understanding of the mechanisms of toxicity. Rodents are commonly employed in developing a new generation of drugs to target the causes rather than the symptoms of PD [287]. To date, most of the work on MPTP using mice demonstrates that cell death following acute exposure of the toxin occurs in two phases: initial cell death commences within hours after the insult [166], peaking at around 5 days [199] with the second phase lasting ≈ 21-28 days [36, 95, 192]. Like most animals models, the acute MPTP model does not reflect all aspects of the human disease. For example, acute intoxication with MPTP does not result in inclusions within the SN [118, 395] which suggests that DA neurons are dying before protein aggregation can occur. More recently, rodent models that employ chronically administered MPTP, exhibit intracellular α-synuclein-positive electron dense inclusions [118]. Chronic MPTP administration is shown to up regulate the expression, the nitration and also the aggregation of α-synuclein within the SN [414]. Conversely, animals that lack α-synuclein are spared and have been shown to be protected against the toxic effects of chronic MPTP treatment [116]. This suggests that the uptake of MPTP is rapid due to high affinity DA re-uptake transporters. The conversion of MPTP into MPP^+^ by Monoamine Oxidase B (MAO-B) allows the reduced form to affect mitochondrial metabolism eventually leading to the inhibition of complex I and release of *cytochrome c* from the inner membrane of the mitochondria [237]. This model is one of the keystones of the oxidative stress hypothesis for PD.

### 2.3 Protein Aggregation

The failure to clear damaged and cytotoxic protein aggregates is often a common feature of most neurodegenerative disorders including PD. Proteasome dysfunction has been observed in dopaminergic neurons in PD patients [278] and cortical neurons in AD cases [208]. Mutations in genes encoding proteins within the ubiquitin proteasome system (parkin and UCH-L1) further link this pathway to PD. Functional and structural deficits within the proteasome pathway prevent protein clearance, leading to proteolytic stress [278]. There has been growing speculation that proteasome dysfunction *via* proteolytic stress could be the underlying mechanism of LB formation and neurodegeneration in the SN in both familial and sporadic PD [278-282].

As the cellular machinery for protein degradation or repair becomes overwhelmed in the disease, failure of this pathway leads to protein accumulation and ultimately cell death [415]. It is clear that an abundance of damaged proteins which occur through oxidation or nitration processes can lead to proteasome inhibition as seen in sporadic PD cases. Proteasome inhibition seems to initially affect DA neurons by modifying DA re-uptake [281, 299]. In cell culture models, the presence of oxidative products such as hydrogen peroxide (H_2_O_2_) and peroxynitrite (ONOO^-^) inhibits the activity of the 20S proteasome [339]. Excessive levels of oxidative modifications to the proteasome causes a decrease in proteolysis, decreases in solubility, stabilization of proteins *via* cross-linkages and protein aggregation [142, 314]. However, what remains unclear is whether the precise nature of LB formation is a systemic defense mechanism acting as a neuroprotectant or is a result of the disease.

### 2.4 Oxidative Stress and Parkinson’s Disease

Neurodegeneration is a multifaceted process and the mechanisms that result in cellular death are linked to events that cause oxidative stress. Although there is much evidence in favor of this hypothesis; there is no definitive study. In the current section, the discussion focuses on: the markers of oxidative stress that have been observed in PD; how the cell becomes stressed; what oxidative damage does to cells; and cellular defense mechanisms to help overcome oxidative stress. 

Oxidative stress is a key pathological process that is common to all neurodegenerative diseases. While oxidative stress occurs over the entire brain, the nigral environment appears to be more sensitive to oxidative stress [10, 62, 92, 104, 139, 142, 164, 259, 358, 436]. Although loss of DA producing neurons is seen in both normal aging individuals and PD cases it has been hypothesized that these catecholaminergic neurons are particularly susceptible to oxidative stress [67, 68, 119].

Oxidative stress markers have been observed in SN specimens in many PD studies. Oxidative biomarkers shown to be elevated in PD brain tissue include: 4-hydroxy-2-nonenal (HNE) [437], protein carbonyls [9] and 8-hydroxyguanosine (8-OHG) [445]. Reduction and protection of remaining neurons from oxidative stress is currently the focus of the pharmaceutical industry with the aim of developing new classes of therapeutic agents for PD. In this review, reactive species (RS) will be used as a broad term of all reactive species including nitration species. In normal healthy aerobes, O_2_ is utilized for cellular respiration processes by the mitochondria [164]. Mitochondria are a major source of RS production as leakage of electrons from the electron transport chain are slowly accepted by O_2_ producing free radicals including the superoxide radical (O_2_^•^). Superoxide is chemically inactive however, if allowed, substantial production of this species can cause biological damage. The covalent bonding of O_2_^•^ to nitroxide (NO) forms peroxynitrite (ONOO^-^) which under physiological conditions can be very reactive and can oxidize lipids [338], DNA [391] and proteins [40, 95, 257]. Superoxide can also be converted to other RS products such as H_2_O_2_ and hydroxyl radicals (OH^•^) which may in turn attack other macromolecules such as proteins, lipids, sugars, and polynucleotides which are susceptible to oxidative damage. The abundance of O_2_^•^ and ONOO^-^ can accelerate the oxidization of iron or copper sulfur clusters found in many proteins that require these clusters for functional activity [209, 210]. Superoxide has been demonstrated to “leach” iron from metalloenzymes, increasing the free iron content [209]. The presence of these free redox active metals can in turn accelerate RS production. Excessive accumulation of H_2_O_2_ and O_2_^•^ in the presence of catalytically reduced transition metals such as iron, copper and manganese can generate a potent hydroxyl radical species OH^•^ *via* Fenton chemistry (1).

(1) $${\text{Fe}}^{2+}+{\text{H}}_{\text{2}}{\text{O}}_{\text{2}}\to {\text{Fe}}^{3+}+{\text{OH}}^{•-}+{\text{OH}}^{-}$$

Superoxide as well as ascorbic acid and thiols can rereduce oxidized metals *via* the Haber-Weiss reaction to produce OH^•^ from H_2_O_2_. Purines and pyrmidines in DNA and RNA can be attacked by the hydroxyl radical, producing irreparable breakages and oxidized RNA products such as 8-OHG [107, 309, 356].

Radicals can remove H^+^ from polyunsaturated side chains found in membrane lipids. The effect of lipid peroxidation is dramatic, causing disruption of membrane fluidity allowing the leakage of molecules that normally cannot cross the membrane. Metal ions can also rapidly induce lipid peroxidation by continually removing H^+^ in fatty acid side chains resulting in toxic hydrocarbons and aldehydes (malonaldehydes and 4-HNE). Byproducts of lipid peroxides such as HNE have a high affinity for and inactivate integral proteins such as Ca^2+^ and K^+^ ion channels and receptors [54] and glutamate transporters leading to a greater excitatory effect and possible excitotoxicity [39, 323].

**Fig. (1):**
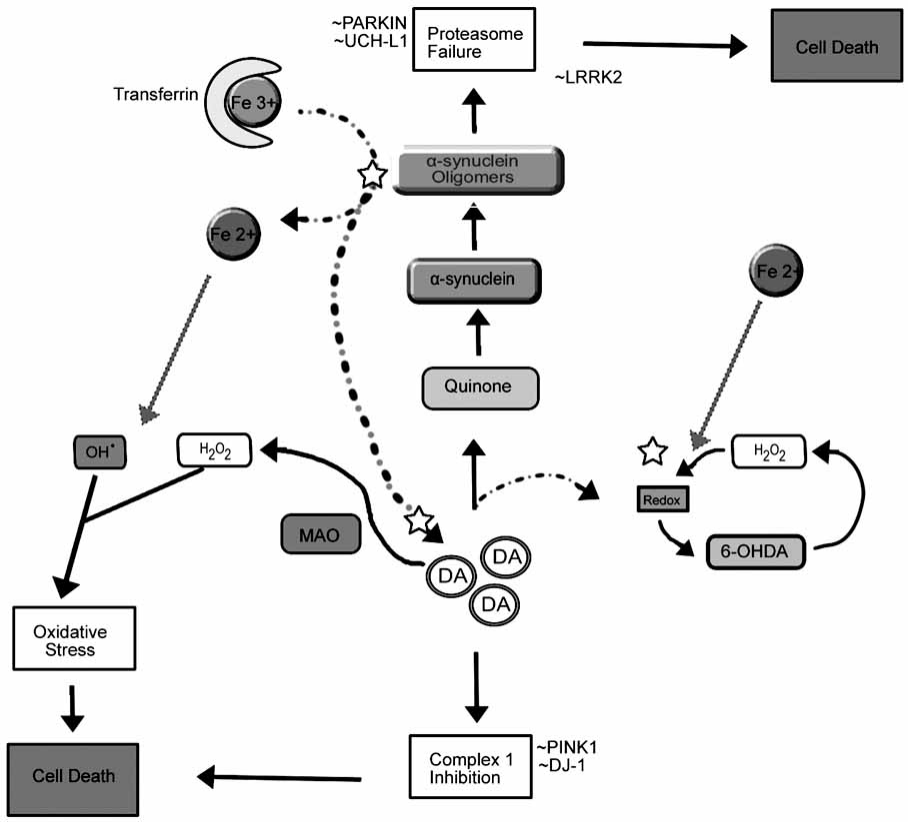
An Oxidative stress model suggesting the roles of Iron (metals), α-synuclein and Dopamine (DA) in the propagation of neurondegeneration in PD (modified from [41]). The stars indicate the points on the pathways that MPAC compounds can influence the reaction.

DA is highly reactive and produces RS through two pathways. In the first instance, DA can undergo oxidation in the presence of molecular oxygen to form H_2_O_2_, 6-OHDA, quinone intermediates and O_2_^•^ (see Fig. **1**). The generation of oxidized DA metabolites can further feed into the redox cycle, leading to the amplification of RS products eventuating in neuronal death. *In vivo* models have shown that DA oxidative metabolites, in particular 6-OHDA, can induce toxicity by generating RS and initiating caspase activation [11]. Secondly, DA can also be broken down by MAO-B through deamination to produce H_2_O_2_ [67]. Further redox cycling can be driven by excess O_2_^•^ which reacts with both DA and metal ions to produce more RS.

It is inevitable that aerobic organisms produce RS. RS can be cleared under normal conditions by the anti-oxidative enzymatic activity of catalase, Copper/Zinc superoxide dismutase (Cu/Zn SOD) or glutathione (GSH) peroxidase. The importance of these enzymes has been demonstrated in transgenic and knockout mice [130, 405]. Cu/ZnSOD, catalase and GSH peroxidase transgenic mice have been tested in both the MPTP and the 6-OHDA lesion models and data demonstrate that there is increased susceptibility to oxidative stress in the absence of these protective proteins [218, 443] while over expression of these proteins result in increased protection [335]. Anti-oxidant systems are essential for defense against cellular endogenous or exogenous oxidants. A decrease in total GSH has been reported in PD *post mortem* SN tissue [63, 322, 365, 374]. The homeostatic balance of RS production to anti-oxidative mechanisms is shifted as the cell becomes burdened and stressed. Elevation in RS [62, 64, 119, 177, 202, 436, 437] and perturbation of anti-oxidative mechanisms ultimately leads to cellular death of individual cells and it is widely hypothesized that there is a feed forward system that leads to progressive neurodegeneration of the nucleus [12, 353]. A decrease in total GSH has been reported in PD *post mortem* tissue [63, 365] where the decrease has been observed specifically within the SN [63, 322, 365, 374]. Neurons seem to be more susceptible to oxidative damage as (i) neuronal membranes are rich in polyunsaturated side chains which are freely attacked increasing the fluidity of the membrane (leaky membrane), (ii) these cells express small amounts of anti-oxidant enzymes such as catalase, GSH peroxidase and Cu/ZnSOD compared to other cells in the body. Much effort has thus gone into the therapeutic intervention of these oxidative processes.

#### 2.4.1 Metals in PD

Transition metals have been implicated in many neurological diseases such as Alzheimer’s disease (AD) [49, 274, 426], Multiple Sclerosis (MS) [242, 284] as well as PD [19, 20, 90, 92, 259, 375]. Aberrant brain metal levels have been associated with normal aging and a variety of diseases however this is still debated [19, 258].

Dopaminergic neurons seem to be highly sensitive to oxidative stress providing a potential link to environmental exposures of metals and PD susceptibility. Epidemiological literature has assessed the potential risk of developing PD when exposed to certain metals such as iron, manganese and to a lesser degree, copper. The risks reflect the potential of redox active metals such as iron and copper acting as catalysts to drive oxidative stress.

It is thought that dysregulation in metal ion homeostasis acts as a potential catalyst to further produce RS as previously mentioned. The current section further discusses how metal ions and in particular iron and manganese are associated with PD.

##### 2.4.1.1 Iron

Iron is required for numerous critical biological processes. These processes are involved in cellular respiration pathways, acting as the central core for metallo-proteins, neurotransmission and myelination. The balance of iron content is essential, as excess iron is highly toxic to cells, seen in some neurological (PD, AD and MS) and peripheral diseases (Haemachomatosis and Friedrich’s Ataxia).

##### 2.4.1.2 Iron Distribution in the Brain 

Dynamically, the brain has the capacity to adapt to abnormal iron levels and redistribute iron to regions of high requirement. An average adult brain has approximately 60 mg of non-heme iron, with some cerebral regions such as the SN, GP, caudate nucleus and putamen retaining the highest level of iron [19, 267]. Most of the iron in brain is found specifically in neurons as iron is a co-factor for many enzymatic reactions (such as the production of DA). Tyrosine hydroxylase (TH) is a non-heme iron enzyme which uses molecular oxygen to hydroxylate tyrosine to form L-DOPA [153]. The brain acquires the critical level of iron necessary for human adult stores through breast milk [219]. At birth, very little iron is present within the developing brain. However,  cerebral iron levels rapidly increase specifically during the early years of life. Approximately 0.3 mg/L of iron is found within human breast milk [118] and uptake of iron occurs within the first 12 to 18 months of human life. In rodents, the uptake of cerebral iron occurs during the 3^rd^ week post partum [79] and during these critical developmental periods; the brain requires iron for normal neurological maturation.

The manner in which iron is transported into the brain is a complex process which is still not well understood. Circulating iron, once oxidized to its ferric state by the serum protein ceruloplasmin cannot readily cross the blood brain barrier [294]. For iron to be transferred across the BBB, the endothelial cells lining the cerebrovasculature require a transfer protein Transferrin (Tf) to which the complex (Tf–iron) binds to transferrin receptors (Tfr) found on the luminal side of the membrane. The complex enters the cells *via* endocytosis and is transported to various cell types within the brain such as neurons, glia and oligodendrocytes. This process is highly regulated by the abundance or the deficiency in Tfr and the Tf-iron complex. Transcriptional regulation of iron binding proteins transferrin and ferritin is controlled by iron regulatory proteins (IRP) which bind to iron responsive elements (IRE) on RNA to alter the expression levels [56, 212]. IRP2 knockout mice develop motor deficits (tremor and bradykinesia), progressive neurodegeneration and increases in ferritin levels within affected neurons [236].

Ferritin is a common iron storage protein within the brain and is expressed in microglia, oligodendrocytes and neurons [71, 190]. Ferritin possesses ferroxidase activity that catalyzes the conversion of ferrous iron to ferric iron. Ferritin acts to reduce the amount of free cytosolic iron by catalyzing iron to its non reactive or ferric state [154]. This mechanism prevents iron from being available to participate in Fenton chemistry and generating RS.

Neuromelanin (NM) is another storage protein with a high affinity for iron [441, 442] and is localized within regions of high metabolic turnover such as in DA producing neurons in the SN and the noradrenalin neurons in the locus coeruleus [42, 349]. Catecholaminergic neurons found in primates contain NM, as in humans, but are less visible and are not localized within the SN [263]. Rodents possess DA and noradrenalin neurons, but these neurons do not appear to contain NM [17]. NM is a byproduct of catecholamine metabolism and is synthesized from quinone intermediates when cytosolic DA is in excess [390]. It has been suggested that NM is a neuroprotectant, preventing degeneration of nigral neurons by binding transition metals and other DA oxidative products that are abundant in the SN. It has been suggested that in situations of high iron overload, NM retains iron or other heavy metals within dihydroxyindole groups on its chemical backbone. The functional role of NM upon its binding to heavy metals is beneficial, by sequestering and preventing any free metal ion from participating in Fenton chemistry. This role allows the SN neurons to tolerate a higher iron load than other neurons within the brain that do not possess this protein. However, during the progression of PD; the NM molecule reaches saturation. Once the NM iron-chelating capabilities are saturated the iron-saturated molecule could be available to create RS [102]. NM appears to be an important buffering molecule that is involved in DA neuron protection and possibly vulnerability.

##### 2.4.1.3 Dysregulation in Brain Iron and Neurodegeneration 

There is an overwhelming consensus that iron accumulation has a pathogenic role and this has been seen in many neurological diseases. This highlights the use of metal altering drugs as a potential therapy for PD (see later section). Iron dys-homeostasis is highly evident in post mortem PD brains [157]. The association of elevated iron found in parkinsonian brains may be linked to age related changes in redox active metals. Post mortem studies have shown that nigral cells are associated with elevated levels of both ferric and ferrous ions within the SN in severe cases of PD [90, 126]. Furthermore, iron has been shown to accumulate in the SN in animals following 6-OHDA and MPTP lesion [112]. Iron is potentially pathogenic as it is a highly redox active metal and can participate in metallo-redox reactions (as discussed in section 2.4). Changes in iron homeostasis which particularly increase the labile iron pool potentially promote neuronal toxicity by catalyzing conversion of a less reactive inert species (H_2_O_2_) to the highly reactive hydroxyl radical. Currently, it is unknown if the dysregulation in iron homeostasis may be a primary or a secondary cause of PD, however, many genetic and biochemical studies have suggested that iron accumulation may be a primary event.

Proteins that regulate iron are disrupted in PD patients [21, 90, 112]. Mutations in iron regulating or binding proteins have been reported in other iron overloading diseases with overlapping clinical features to PD such as neuroferritinopathy, Friedrichs ataxia, and haemochromatosis. Increased levels of the iron storage protein ferritin have been shown in PD patients [21, 90]. Transgenic animals that express increased ferritin levels within dopaminergic neurons in the SN have shown age-related progressive neurodegeneration, loss of axonal projections and decreased DA in the striatum together with loss of spontaneous movement [76, 206]. Gene knockout mouse models of haemochromatosis showed severe deficits in motor performance [147] attributable to the CNS.

Point mutations near the iron binding site of the TH molecule have been found in cases of L-DOPA responsive PD and Segawa’s syndrome that results in TH deficiency [152]. Furthermore, iron plays a critical role in electron transport and the metabolism of various neurotransmitters including DA, norepinephrine and GABA as well as in DA D2 receptor function [373].

Direct bolus intranigral injection of iron has shown to increase the levels of iron within the SN [25, 424] and results in severe loss of nigral cells associated with reduced DA levels in the striatum and deficits in locomotor activity. The risks of high dietary iron at a given critical vulnerable stage have been highly correlated to late onset development of PD [124, 125, 205]. During brain maturation, dietary iron induced in rat and mice neonates resulted in patterns of PD like behavior and nigral cell degeneration in adulthood [124, 125]. Excess dietary iron post partum, increases the levels of iron within the whole brain but to the greatest extent in the GP and the SN [330]. The transport of iron to the brain peaks between birth and 21 days *post partum *in neonatal mice, with little entering the brain after that time. Feeding a high iron diet generates characteristics of PD later in life in these animal models [125, 373]. Feeding of 20000 ppm for 12 weeks causes behavioral dysfunction and severely affects the latency in motor activity [373]. Mice that are exposed to a high iron diet during neonatal development have an increased susceptibility to oxidative stress and a reduction in TH positive cells at 24 months of age [205]. Rodents deficient in neonatal iron are deficient in brain ferritin [167], and have reduced cognitive functioning [84, 148, 330]. This evidence notwithstanding, the direct effects of iron during early human development and its contribution to a potential increase in susceptibility to PD is a controversial topic that has not been fully addressed to date.

##### 2.4.1.4 Copper

The interrelationship with iron and copper has been discussed in brief in papers [90-92]. Few studies have investigated copper and its role is yet to be established in PD.

##### 2.4.1.5 Manganese

Manganese toxicity, also known as *manganism* has been characterized in miners following long term exposure to manganese ore [187-189] and occupational studies have correlated welders with a high prevalence of parkinsonian like symptoms, such as abnormalities in gait and speech [337] following exposure to high levels of manganese from the welding rods. One clinical difference between PD and *manganism* is that *manganism* produces dystonia [16]. This difference is attributed to the GP being primarily affected in *manganism* [363] with only minor damage to the SN [432]. These commonalities suggest that manganese and iron affect the dopaminergic system where they accumulate within these regions. Following a 6-OHDA lesion to the SN significantly higher levels of manganese were detected in the GP, SN, amygdala, hypothalamus, and hippocampus [394]. It has also been suggested that the increase in manganese content found in the SN after a 6-OHDA lesion directly contributes to the accumulation of iron levels within the SN [394]. Like iron, manganese can also participate in redox chemistry and generate RS causing cell death [318]. Evidence demonstrating that manganese affects dopaminergic neurons *via* oxidative stress damage to DNA of these neurons [310] include elevation of DA oxidative metabolites [358] and induction of apoptotic pathways in cultured cells [88]. Furthermore, manganese can reduce anti-oxidants such as glutathione (GSH), catalase and thiols [87, 246].

##### 2.4.1.6 Aluminum

The hypothetic role of aluminum and its pathogenic role seen in neurodegenerative diseases, has managed to survive in the neuroscience field in spite of ambivalent support. Aluminum in drinking water was linked to AD, ALS and PD until it was discovered that the epidemiological studies were inadequately designed and that aluminum is poorly absorbed by the digestive system [115]. The presence of aluminum is quite low within living organisms, but is highly abundant within the environment. Aluminum can be detected in foods, pharmaceutical agents (antacids) and can also be found in drinking water. Aluminum has been generally considered biologically inert. In spite of this, the toxicity of aluminum has been well established. Few epidemiological studies have made connections between aluminum and neurodegenerative diseases such as AD [138, 155, 161, 325, 332, 345], ALS and PD [26, 369]. Currently, there is no data that suggests aluminum (a trace element) is essential for bodily function. While no reliable measurement of total body aluminum content is available, the body’s acquisition of aluminum is largely by ingestion [292, 396, 397]. Microdialysis studies have shown that aluminum can cross the blood brain barrier *via* the aid of a transporter mechanism which included an iron carrier molecule transferrin [293]. Historically there has been concern, concentrations of aluminum in drinking water have been investigated as a potential factor to give rise to AD [115, 138, 345, 379]. Whilst there is evidence which evinced no relationship [268, 425], a considerable number of studies have attempted to defined this link [345]. There is pathological data showing elevated aluminum in the spinal cord and the hippocampus from ALS and PD with dementia [137, 216, 326, 329] and in tissues from PD patients [151, 180]. However the elevation in aluminum seen in these diseases may not be related to dietary intake.

The biological effects of artificially increased aluminum in experimental animals [211, 216, 379] is to cause pathological changes; including neurofibrillary tangles which closely resemble those in AD, and neuronal loss [137, 211, 216, 224, 325, 406]. Interestingly, these aluminum treated animals showed signs of loss in motor functions found in the hind limbs [216] which suggests that aluminum affects other modalities in the CNS. Together with iron, aluminum has been reported to be significantly concentrated in melanized granules within the SN [151, 180]. The presence of aluminum and iron accelerate the formation of lipid peroxides [159] and thus act in a synergistic fashion. Aluminum can participate in chemistry that induces changes in membrane fluidity that can facilitate lipid peroxidation [283]. While aluminum is redox inactive, a 3 week exposure of rats to aluminum salts induced increased levels of RS in cortical regions [43]. The speculation that aluminum can potentiate and generate a pro-oxidative environment has been supported by studies suggesting that aluminum can trigger inflammation responses [51]. Interestingly, Mendez-Alvarez and colleagues (2002) found that in a 6-OHDA animal model, the presence of aluminum reduced the OH^•^ production and attenuated the neurotoxic effects of the 6-OHDA lesion [285] by the prevention of lipid peroxide formation. It was concluded that the effects of aluminum accumulation could be by binding to 6-OHDA preventing the interaction between 6-OHDA and hydrogen peroxide [285], thereby reducing the toxic effects of the 6-OHDA.

Aluminum toxicity has recently been revisited because of its effect *in vitro* and in animal models but there is little evidence on aluminum has any involvement in PD and it is therefore is not considered as a therapeutic target at this stage.

### 2.5 Integration of Current Models for Therapeutics

Dopaminergic neurons appear to be generally under high oxidative loads and are thus suspected of being susceptible to neurodegeneration. Iron, DA and α-synuclein, all co-localize to the SN and these factors are implicated in the etiology of PD. However it has been substantiated that none of these elements alone is sufficient to cause the observed chronic neurodegeneration. Elevated iron and DA in the presence of increased or altered metabolism of α-synuclein, may act synergistically to propagate a series of reactions that result in destruction of SN neurons. To address potential new pharmacological intervention therapies for drug development, the observed changes in metabolism that result in PD must be considered.

The highly favorable oxidative stress environment for DA interaction with α-synuclein and iron resulting in RS-mediated toxicity and protein aggregation is one of the most likely mechanistic explanations for PD (refer to Fig. (**1**)). Singular constituents of this model such as DA, α-synuclein or iron alone are not capable of emulating PD-like neurodegeneration. In this section of the review several therapeutic targets are highlighted and discussed.

DA has been implicated to play a role in the neurodegenerative cascade in PD as it is susceptible to oxidation (discussed in section 2.4). Following a partial lesion of the dopaminergic SN neurons the remaining neurons compensate by increasing the amount of DA produced as well as increasing the rate of DA turnover [383]. In surviving neurons, the DA turnover rate is increased by the deamination of DA by MAO-B thus increasing the production of H_2_O_2 _and oxidative stress metabolites [67]. DA is readily auto-oxidized through metallo-redox reactions to produce the neurotoxin 6-OHDA and further oxidized into quinone intermediates to generate O_2_^•^ [202] (See Fig. 1). As 6-OHDA is analogous to catecholamine neurotransmitters, it may be taken up by the corresponding reuptake transporters [335] resulting in rapid loss of nigral neurons by activation of caspase pathways [99]. Direct injection of 6-OHDA into the SN or the medial forebrain bundle is widely used in animal models to induce parkinsonism [131, 194, 439].

Ferrous ions catalyze the formation of oxidized DA, generating free radicals but also converting oxidative metabolites such as 6-OHDA into melanin [103]. Manganese can also participate in the oxidation of DA leading to the generation of RS [310] which implements further redox (active or inactive) metals that can be substituted in this reaction.

The familial A53T α-synuclein mutant raises cytosolic DA which increases the available substrates for this cascade [203, 254, 296]. Over-expression of DA metabolic genes such as α-synuclein accelerate the rate of DA re-uptake *via* DAT therefore increasing DA vulnerability [203, 392], further RS production and DA induced apoptosis [392]. 

α-Synuclein mRNA expression has been seen to be only elevated within the SN in later stages of PD [61, 158, 414]. This effect could be due to the relationship between DA levels and α-synuclein that facilitate the transmission of DA. Synaptic regulation is shown to be severely affected when α-synuclein expression is altered [412]. In the presence of α-synuclein, DA-α-synuclein adducts form through the stabilization of protofibrillar structures [73]. This is exacerbated by the presence of iron which also triggers aggregation of α-synuclein by changing the protein conformation structure from helices into an unfolded beta-sheet structure [313]. The fibrillated protein is the major component in LB [154]. Formation of protein aggregates is likely to be by the direct association between metals and α-synuclein.

It is interesting to note that the familial mutations of α-synuclein are localized within the metal binding site of α-synuclein and each of these mutations affect metal interactions with the protein [29]. A metal responsive element has been demonstrated in the promoter located within the 5’ Untranslated Region (UTR) of the α-synuclein mRNA transcript [129]. This suggests that α-synuclein may be a metallo-protein which can be manipulated to help treat PD.

Metal ions in the presence of DA directly cause α-synuclein protein aggregates [53] and modifications of α-synuclein protein occur in the presence of RS generated by Fenton chemistry. Post translational modifications such as phosphorylation, glycosylation, oxidation and nitration of α-synuclein promote protein aggregation [139]. Oxidatively modified α-synuclein and aggregates stabilized through the dityrosine cross-linkages are observed in LB [378]. Through further oxidization of α-synuclein, sequential oligomerization is enhanced by the presence of copper [315]. Nitration of α-synuclein promotes the formation of high order oligomerization [303] which may perforate vesicular membranes such as DA storage synaptic vesicles, resulting in leakage of DA into the cytoplasm [255]. In addition, aggregated α-synuclein may over-stimulate TH activity causing overproduction of DA, propagating a feed forward degenerative cascade [53, 324]. α-synuclein mutants as well as increased levels of normal α-synuclein generate RS [239], which is accelerated in the presence of DA and increases susceptibility of cells to oxidative stress [203].

Mutated α-synuclein has a greater propensity to polymerize *in vitro* and α-synuclein transgenic mice develop age-dependent intracellular α-synuclein inclusions within aberrant areas of the CNS. Further, the expression patterns of mutated human α-synuclein and the location of inclusions, in the various transgenic mouse models, appear to be influenced by the different promoters utilized [270]. Wild type α-synuclein has a half life of 48 hours while the A53T mutation has an approximately 50% longer half life [245] suggesting that these mutant proteins are selectively preventing degradation *via* the lysosome-mediated pathway [78].

Oxidative stress (e.g. from mitochondrial inefficiency, anti-oxidant depletion, or transition metal perturbation) provides conditions under which DA may interact aberrantly with α-synuclein and iron resulting in the generation of H_2_O_2_, oxidation of DA to toxic intermediates such as 6-OHDA, and aggregation of α-synuclein. A self-propagating cascade is engendered as oxidative products of these reactions create conditions for further local elevation of DA and generation of metal mediated RS. Therefore subsequent sections discuss how intervening therapies which target these interactions may be capable of modulating the disease.

## 3 CURRENT TRENDS IN THERAPEUTICS

Alleviation of parkinsonian symptoms and functional disability is the principal goal of PD management in clinical practice. Most patients in early stages of idiopathic PD will improve in response to medications that are directed at correction of the hypo-dopaminergic biochemical deficit and enhancement of dopaminergic neurotransmission. This approach constitutes symptomatic therapy of PD, but the majority of PD patients will gradually deteriorate. It is thought that an ongoing apoptotic death of dopaminergic neurons in SN underpins this relentless natural history of PD. Presynaptic dopaminergic terminals in the basal ganglia release vesicular DA on demand and also carry out DA reuptake *via* the DAT system. Dopaminergic neurons of the SN receive innervation from the basal ganglia, thus creating a complex feedback loop. This illustrates the role of dopaminergic neurons in biochemical processes and also emphasizes that dopaminergic neurons are intimately incorporated into neural circuits. Neuroprotective therapy sets out to rescue the apoptotic dopaminergic neurons in SN. Neuroprotective therapy still remains mostly an experimental approach, but putative neuroprotective drugs may alter the relentless course of PD. Detailed synopsis of current symptomatic treatment is out of the scope of this review and is dealt with elsewhere [185, 186]. In this section we summarize only the current most common therapeutic pharmacological strategies and focus on some experimental neuroprotective therapies currently undergoing human trial.

### 3.1 Symptomatic Therapy

#### Levodopa

At present, Levodopa (L-DOPA or 3, 4-dihydroxy-L-phenylalanine) is the most useful drug for symptomatic treatment of idiopathic PD. Unlike DA, L-DOPA crosses the BBB. After oral administration L-DOPA is taken up by the dopaminergic neurons and converted into DA by the enzyme Aromatic Amino Acid Decarboxylase (AADC). 

L-DOPA effectively alleviates PD symptoms in the early stages of disease. The current “storage hypothesis” holds that at this stage of PD the available dopaminergic neurons and pre-synaptic dopaminergic terminals maintain the capacity to process exogenous L-DOPA and carry out physiological handling of synthesized DA [183, 304-306]. It has been suggested that the benefits of L-DOPA wear off with disease progression and ongoing death of dopaminergic neurons [238]. This view may be misleading due to the inability to discriminate against the treatment effects and the natural progression of the disease. According to the “storage hypothesis”, in the absence of dopaminergic neurons L-DOPA is metabolized into DA by neural cells that lack “dopaminergic machinery”. As a result DA release becomes pulsatile rather than continuous and eventually leads to post-synaptic changes and development of motor complications [5, 55, 429].

At present there is some evidence that L-DOPA can be neuroprotective to dopaminergic neurons. The Early versus Late Levodopa study (ELLDOPA) indicates some neuroprotection all be it with diminishes striatal innervation [111]. The DATATOP study also suggested that L-DOPA slowed the rate of disease progression [1, 57, 183]. In contrast, *in vitro* experiments suggest that L-DOPA accelerates degeneration of residual dopaminergic neurons through enhanced oxidative stress. However, L-DOPA was not toxic to dopaminergic neurons *in vivo* in experimental rodents. Recent human trials presented unequivocal evidence that L-DOPA treatment did not cause clinical deterioration over a period of 40 weeks compared to the placebo [69, 110, 229, 298, 385, 418]. However, the potential long-term effects of L-DOPA on dopaminergic neurons remain unclear.

#### Direct Agonists of Dopaminergic Receptors (or Dopamine Agonists)

The rationale for developing this class of drugs was the delivery of continuous stimulation of dopaminergic receptors, thought necessary to prevent development of motor fluctuations in long-term. This approach was put forward as an alternative to L-DOPA treatment, based on the hypothesis that L-DOPA treatment set pulsatile stimulation of postsynaptic dopaminergic receptors and promoted development of motor fluctuations.

Numerous *in vitro* and *in vivo* laboratory studies have shown neuroprotective potential of dopaminergic agonists that can be mediated *via* several mechanisms including free radical scavenging [149, 221], and anti-oxidative properties [352, 453].

Data from human trials are not conclusive as to neuroprotective properties of DA agonists in PD patients, chiefly because it is very difficult to discriminate between symptomatic and putative neuroprotective effects in the settings of clinical trials and requires a sophisticated approach to the design and analysis of the study. However, current experience with PD patients suggests that the impact of direct DA agonists on the natural course of PD may not be of a clinically meaningful magnitude [6].

### 3.2 Drugs with Dual, Symptomatic and Neuroprotective Effect

#### MAO-B Inhibitors

There are currently two selective irreversible MAO-B inhibitor drugs approved for clinical use, rasagiline (Azilect) and selegiline (Deprenyl). Two isoforms of MAO have been identified, A and B. In the human brain, MAO-B is the predominant isoform responsible for the breakdown of DA. Selective inhibition of MAO-B results in the elevation of synaptosomal DA concentrations. The primary rationale for MAO-B inhibition in PD is enhancement of striatal DA through inhibition of DA metabolism and the role of MAO-B inhibitors in symptomatic treatment of PD has been well established [317]. 

Interestingly, both selegiline and rasagiline possess potent neuroprotective and anti-apoptotic properties that are not related to MAO-B inhibition. This effect has been demonstrated *in vitro* using primary cultures of cortical neurons; both drugs enhanced survival of dopaminergic neurons. Neuroprotection has been demonstrated *in vivo* in rodent models of Parkinson’s disease. It has been proposed that stabilization of mitochondrial membranes, enhancement of intracellular anti-oxidant systems and induction of pro-survival genes underlies this effect (for review, see www.rasagiline.com).

Recently, the interest in neuroprotective properties of MAO-B inhibitors has been sparked by the study showing that Selegiline slows progression of PD symptoms by about 35% over 5 years [317]. This suggests that there may be neuroprotective effects on the nigro-striatal system. Rasagiline has only recently gained FDA approval, but preliminary results have been promising [366].

### 3.3 Cell Based Therapies

The neuropathological and neurochemical alterations of the dopaminergic nigro-striatal system are responsible for the major symptoms of PD (see above section). This constitutes the premise of DA cell-replacement therapy, whereby introducing DA-producing cells into the parkinsonian brain might replenish the diminishing levels of DA and alleviate or cure PD. 

Over the last 20 years there has been an enormous research effort in this field of neuroscience. Swedish neuroscientists pioneered transplantation experiments in the mid-70’s and early 80’s [33, 36, 108]. In early transplantation experiments the grafts of DA-producing cells were placed into the striatum because this approach yielded best survival of the grafted cells with subsequent dopaminergic reinner-vation of the basal ganglia [31, 32, 34, 35, 106, 133]. In parallel, the demand grew for the sources of DA-producing cells. Traditionally, fetal ventral mesencephalic tissue has been used for grafting because this region of the developing brain contains precursors of dopaminergic cells which differentiate into functional DA-producing cells *in vivo*. Ethical issues essentially preclude large-scale use of the fetal-derived ventral mesencephalic tissue. An alternative approach has been developed, whereby embryonic stem cells or committed neural precursors can undergo directed *in vitro* differentiation into DA-producing cells, these are then harvested and used for transplantation [37, 248, 346, 387].

Several groups in Europe reported that PD symptoms improved following grafting of the fetal mesencephalic tissue into the putamen or head of caudate area of PD patients [162, 163]. Based on the promising preliminary results, NIH funded the first prospective, double-blind, placebo-controlled trial in which 40 PD patients received fetal mesencephalic transplants or placebo operations [126]. The functional improvement of participants was assessed 12 months following grafting using the Unified Parkinson’s Disease Rating Scale (UPDRS). Fetal mesencephalic transplants induced statistically significant improvement in a cohort of patients under the age of 60. Long-term follow-up of the participants revealed five patients who underwent transplantation developed dystonia and dyskinesia. Another prospective, 24-month, double-blind, placebo-controlled trial of human fetal nigral transplantation [311] failed to detect significant differences between grafted and placebo groups. The incidence of dyskinesias was high in this study affecting almost half of the patients that had received mesencephalic transplants.

The two human trials are commonly designated as “proof-of-concept” studies and the negative result are perceived as compromising the entire concept of the cell-replacement approach. However, several factors have been identified that confound interpretation of the negative results of human transplantation trials and require further clarification: surgical technique needs improvement; non-dopaminergic cells within fetal tissue transplants also have been implicated in post-surgical complications [30, 127, 128]. The current mainstream of research is directed at producing a reliable and standardized population of DA-producing cells such as neural progenitor cells (NPC) that can be used for further transplantation trials.

### 3.4 Neurosurgical Therapies

Neurosurgical interventions have developed symptomatic treatments for motor related disorders particularly for advanced PD patients with ensuing dyskinesias. 

With the increasing knowledge of the neuroanatomical circuitry, surgical treatments such as precision surgical ablation (pallidotomy and thalamotomy) and Deep Brain Stimulation (DBS) are favorable procedures due to the shortcomings of pharmacological therapies. Surgical ablation therapy has been used in many instances until the late 1990’s to reduce severe behavioral symptoms such as bradykinesia, dyskinesia, and rigidity and to some extent resting tremor. Targets for functional neurosurgery include the ventral intermediate nucleus (ViN), STN or the internal Globus Pallidus (GPi). It is though that the reduction of GPi activity through ablative surgery rebalances the inhibitory effect of the abundance in striatal GABA due to the loss of DA production.

In many models [174] and also human parkinsonism, STN and GPi ablation have shown to improve behavioral outcomes associated with the disease. The procedure itself is irreversible, with serious complications that could lead to permanent disability particularly impairment of speech and visual modalities.

Unilateral pallidotomies are still preformed today without any knowledge of the long term effects of the surgery. While results of bilateral GPi lesions are indicative of a reduction in dyskinesia [81], there is an association with increased risk of inherent adverse side effects [81]. Patients with unilateral subthalamic lesions showed pronounced improvement particularly after surgery [321]. However, efficacy was limited in that tremors would reoccur in about 20 % of cases. A current alternative surgical treatment, high frequency DBS, has replaced stereotaxic lesioning. Chronic high frequency stimulation of the STN (*via* ViM) in a pilot study in 1987, yielded some promising results by a reduction in extra pyramidal side effects [24]. This new treatment paved the way for a new type of functional motor disorder neurosurgery without subsequent adverse side effects associated with surgical ablation. In PD, the motor deficits are attributed to increased neuronal activity within the STN and the GPi. The surgery involves an insertion of an electrode attached to a neurostimulator. The neurostimulator sends out electrical signals that modulate neuronal circuitry in target areas in the brain to inhibit the impulses that give rise to motor dysfunction. The electrode is placed into a region to address a particular motor symptom accordingly. DBS is proven to be an efficacious treatment in studies that target regions of both the STN and GPi. These studies revealed a reduction in symptoms such as tremor, bradykinesia and rigidity [217, 388]. The stimulation of either the STN [226] or the GPi [2] resulted in significant improvements in UPDRS motor scales.

The mechanisms of STN-DBS and GPi-DBS are paradoxical and still remain unknown. It was hypothesized that electrical stimulation of the STN and GPi would suppress these structural inputs [101] and would therefore act as a counteractive measure of DA replacement therapy. Conversely, Stefani and colleagues (2005) [384] questioned this mechanism suggesting that STN-DBS increases GPi firing rate and synchronizes the STN activity. In a microdialysis model of PD, cGMP was used as a measure of glutamate transmission and was found to be increased by 6 fold in GPi dialysate [384].

Concurrent electrical stimulation is a reversible procedure with fewer surgical complications compared to its lesioning counterpart. Follow-up studies of bilateral STN-DBS patients showed improvement in motor symptoms [109] suggesting that DBS is a beneficial long term treatment. The DBS is a useful procedure because it allows the stimulation of an affected region without further destruction of brain tissue [160]. At the same time, long term stimulation of the STN could aid in slowing the disease progression. However, this is yet to be confirmed since Hilker and colleagues (2005) [179] established that bilateral STN stimulation did not alter the rate of disease progression.

## 4 NEW THERAPEUTIC STRATEGIES

Current therapeutics for PD is neither curative nor preventative as they only temporarily alleviate some of the symptoms of the disease. Drug intervention needs to aim at halting the progression of PD. Current treatments of PD are successfully improving quality of life but unfortunately largely without the ability to control or reduce the rate of disease progression. An integrative model that combines the putative fundamental aspects of nigral degeneration is needed for appropriate therapeutic targeting to potentially prevent further DA nigral loss.

### 4.1 Gene Therapy

With the current knowledge of molecular characterization of vital genes involved in the neurodegenerative process, several research groups have embarked on using gene therapy to help protect and also repair neuronal damage. Deliverance of protein products is difficult as the BBB limits the transfer to the intended destinations. Genetic manipulation has many advantageous applications with many vehicles aiding in the delivery of the gene target such as viral (lentivirus, adenovirus and herpes virus) or non viral (polyplexes) that can infect cells without inducing inflammatory responses and has the ability to affect both dividing and non dividing cells [105, 277]. Furthermore, the regulatory control of an element that allows the expression of the gene is the primary mechanism for genetic manipulation. Whilst many genes have been uncovered acting as potential ‘players’ in the degenerative cascade, this has allowed the makings of *in vivo* gene therapy promising a new future treatment for PD. The conceptual difficulty in this approach comes from the unknown cause of sporadic PD [400]. Once the cause has been identified, gene therapy may then take greater prominence. While this interventional approach is still a new concept, only a few genes have been trialed in animal models of PD. These gene targets include α-synuclein [123, 171, 239, 350, 451] and Parkin [192, 251, 362, 434].

The use of various neurotrophins in support of the nigral neurons has proved effective in various animal models [59, 70, 225]. The Glial cell line-Derived Neurotrophic Factor (GDNF) has shown the propensity to increase the rate of DA neuronal survival under neurotoxic cell culture conditions [48, 65] and in animal models [59, 70]. Reports of GDNF therapy delivered with an adenovirus have been used in animal models and it was found that GDNF can rescue DA cell loss if administered prior to or shortly after delivery of 6-OHDA [225, 372] or MPTP [222]. Significant improvement in motor behavior is a reflection in significant DA cell recovery of function [421, 450] and correlated with a higher level of DA production in the striatum [421]. These higher levels of DA may occur because of increased TH expression [371]. The results from the various clinical trials have not been so definitive. Lang and colleagues (2006) [232], showed that there was no significant clinical benefit in the UPDRS in a phase II trial that investigated the effect of intra-putamen infusion of GDNF, yet similar studies have shown significant progressive improvements in open-label designed trials [141, 370]. It has been debated that Lang and colleagues (2006) did not adequately take into account the placebo effect, the catheter design or the rate of delivery of GDNF [232]. Further, recalculation of the statistical power showed that the power of the study was unable to investigate the effects of GDNF in PD [191]. In light of these difficulties, GDNF properties of promoting cell survival have not been adequately tested in trial for neuroprotection therapy as this would require a longer period of evaluation and sophisticated study design.

Preliminary data using gene therapy to target the STN instead the SN has showed some promise as a therapy in PD. Utilizing a viral expression system, an enzyme (glutamic acid decarboxylase) that synthesizes a neurotransmitter (GABA) was surgically introduced into the STN of patients with PD [113]. The rationale of targeting the STN instead of the SN (which is a primary target seen in many gene therapy trials) was to functionally increase the production of GABA to decrease the aberrant increase in signals to the thalamus [113]. Significant improvement was reported clinically with the functional restoration of circuitry and improvements in motor behavior. Whilst successful in a pilot phase, the question of the placebo effect seen in many surgeries has not been adequately addressed [82]. The clinical presentation of PD symptoms occurs when at least 70% of nigral cells are lost. Goals of therapeutic interventions must therefore address the recovery and prevent the progressive nature of neuronal death in the SN. GDNF partially fulfils these criteria and is therefore an interesting target. However, many factors need to be considered: i) Regulated controlled delivery of gene products [223]. ii) Transfection is notoriously difficult and inefficient in neuronal cultured systems [422] and growing concerns of random integration posing a risk of insertion mutagenesis. iii) Adverse immune reaction [271] and iv) How chronic delivery of these “foreign” genetic products will be restricted to the correct brain region. Gene therapy still has a promising future and remains in an experimental stage. These factors need to be carefully tested before its emergence as an effective therapy for PD.

### 4.2 Anti-Oxidant Based Therapies

Over the last decade, neuroprotective approaches for PD have been tried in an attempt to slow the rate of disease progression. There have been a number of intervention strategies focusing on decreasing oxidative stress. Anti-oxidants can be naturally found in the diet in the form of vitamins (A, C and E), polyphenols, flavonoids and carotenoids. Interestingly, reports of dietary intake (such as high intakes of saturated fats and cholesterols) could possibly influence the susceptibility of developing PD [172, 173, 201, 253]. Dietary anti-oxidants can be found highly in fruits, vegetables, green/ black teas and red wine [144, 289], it appears that moderate ingestion of these foods results in a reduction in risk of PD [144, 252, 353]. The properties of anti-oxidants possesses is the ability to scavenge for free radicals such as the hydroxyl and the O_2_^•^ radical [52, 89, 94, 165, 348, 398]. RS damage can be prevented by selected flavonoids and related phenols (polyphenols) by directly inhibiting both the formation of RS [52, 165, 398] and they enzymes that produce them [140]. A lot of research to date has focused on the properties of phenols found in tea extracts [348]. These potent anti-oxidants have shown to attenuate the toxic effects of 6-OHDA both in cultured PC12 cells [244] and an animal model of PD [243]. 

The protection with the use of carotenoids and both vitamin C (ascorbate) and E (α-tocopherol) has been seen in cellular based models of oxidative stress [367]. However these findings are inconsistent with the data produced by epidemiological studies. The use of vitamin supplements has been assessed in large cohort studies and found that there was no association with reduced risks of developing PD [447]. Among individuals who have a high intake of foods that were rich in vitamin E showed significant reduction in the associated risks [83, 136, 144]. Other studies had not found this association [12, 353].

Clinical based trials have investigated the use of anti-oxidants in PD patients. One of the first of its kind Deprenyl, and α-tocopherol Anti-oxidant Therapy of Parkinsonism (DATATOP) evaluated the use of these agents in a controlled clinical trial setting [1]. The study revealed that α-tocopherol did not benefit in slowing down or reducing the severity of symptoms of PD. This result has been suggested to be a cause of slow absorption and poor penetration into the CNS [1]. Animal models of PD using MPTP evinced conflicting views on the effects of vitamin E [150, 340], vitamin C and carotenoids [327, 328, 417]. Vitamin E deficient mice have an increased susceptibility to MPTP which severely affected the SN [307]. Dietary intake of vitamin E, C and carotenoids in the form of some foods remains consistent over a lifetime, and should be regarded and used in a staple diet from an earlier age. The supplementations of these vitamins are yet to be convincing as a therapy to be used at a clinical level.

A potential new anti-oxidant agent coenzyme Q10 seemingly has some promise as a therapy in mitochondrial disorders and neurodegenerative diseases. Improvement in patients with mitochondrial defects is seen biochemically and clinically with coenzyme Q10 treatment [46, 47, 308]. In neurological diseases that show mitochondrial deficit as a clinical and pathological feature, treatment with coenzyme Q10 could be of benefit. Serving as a potent anti-oxidant, coenzyme Q10 is a lipid soluble molecule which sits in the inner membrane of mitochondria and transfers electrons in the electron transport chain [404]. The anti-oxidative properties involve the ability to scavenge and inhibit the formation of RS [120, 302, 376]. The neuroprotective effects of coenzyme Q10 is seen in many models of neurotoxicity such as rotenone [291] and MPTP [22, 66]. These studies revealed that in animal lesion models, coenzyme Q10 significantly protected against; loss of TH positive cells in the SN; the depletion of striatal DA and the prevention in the formation of α-synuclein aggregates [66]. 

Phase II PD clinical trial showed a reduction (44%) in motor deficits measured by UPDRS using a maximal dose of 1200mg (per day) [364]. Storch and colleagues (2007) [386] attempted to replicate the earlier study with a withdrawal phase using participants with middle stage PD in a more rigorous study design. The treatment with coenzyme Q10 in this study showed no significant motor improvements at a dose of 300mg a day [386]. It was concluded that dosage is not sufficient enough to have a symptomatic effect at this stage of the disease. Further trials need to explore the protective effects in PD using the anti-oxidant coenzyme Q10 at a high dosage and for an extended period of time.

### 4.3 Therapeutics that Focus on Metals 

There has been substantial research into pharmacological interventions that are involved in the modulation of biometals in neurodegenerative disorders. Iron dysregulation seems to play a vital role in disease pathogenesis in PD patients. These disruptions in the iron homeostatic mechanism observed in PD offer the potential for future therapeutic intervention. Controlling the bioavailability of metals could prevent not only the generation of RS through metallo-redox reactions but also the interaction with other known ‘culprit contenders’ such as α-synuclein (as shown in Fig. **1**).

#### 4.3.1 Therapies which Target Metal-Associated Proteins

As discussed previously in section 2.4.1, ferritin is a protein that regulates iron storage and can potentially remove any free redox active iron that is present within the cell. Transgenic mice that express high levels of H-Ferritin have been shown to effectively protect the further loss of nigral cells in MPTP [207] and paraquat [275] animal models of PD. H-Ferritin possesses a modifying oxidase activity, which sequesters the iron and converts it to the less bioreactive form [205, 207]. This increase in ferroxidase activity reduces the free iron pool preventing its further participation in redox chemistry [207]. 

#### 4.3.2 Metal Chelation Therapies 

The primary mechanism of chelators is to chemically bind metal ions to form complexes rendering the ions less reactive and allow removal of these ions *via* the bloodstream for excretion. Pharmacological chelators such as desferroxamine have shown some promise in modulating metal ions. *In vitro* studies have shown that Desferal intervenes in mitochondrial inhibition by directly enhancing the activation of NADH dehydrogenase [438]. Unfortunately, desferroxamine has poor penetration through the BBB [448]. New iron chelators such as VK-28 were synthesized in order to overcome potential barrier impermeability. VK-28 has been shown to protect nigral cells against 6-OHDA induced lesions [357].

In an induced proteasome dysfunction animal model, chelation therapy with desferroxamine reduced the inhibitory effects on proteasome inhibitors [448]. Iron potentially acerbates the rapid formation of the α-synuclein structure to promote high molecular weight insoluble aggregates (see section 2.4.1). Proteasome dysfunction in this model of PD is relieved by sequestering iron to prevent this aggregation from occurring and protect the nigral cells [448]. Pyridoxal Isonicotinoyl Hyrdazone (PIH) and 2-Pyridylcarboxaldehyde isonicotinoyl Hydrazone (PCIH) share similar potency to desferroxamine, possess high and potent chelating activity, have the ability to cross the BBB and are highly specific for iron overload diseases [178, 341]. PIH and its analogues seem to act in a dose-dependant manner in the immobilization of iron from ferritin and allowing excretion [433].

The complexity of parkinsonism has been highlighted and discussed in this review. An observation of this complexity has lead biochemists to develop bifunctional compounds which have two modes of action. Only recently, new compounds have emerged based on known MAO-B inhibitors. These have been synthesized to possess both neuroprotective effects and iron chelating properties [449]. M30, which has similar structure to that of VK-28, is a hydroxyquinoline which acts as both a selective inhibitor of MAO-A and MAO-B with chelating capabilities similar to that of desferroxamine [15, 134]. Both *in vivo* and *in vitro* models have shown promising effects to both increase levels of DA and prevent further MPTP toxicity [15, 134, 135, 449]. While there is potential to synthesize more ‘multi-functional’ compounds, however there is a need to develop a more clinical based approach and evaluate the effects of these drugs both at a physical and biochemical level.

#### 4.3.3 Metal Protein Attenuating Compounds (MPAC) 

Metal protein attenuating compounds (MPAC) may offer future therapies for PD. Clioquinol (5-chloro-7-iodo-8-hydroxyquinoline, CQ) is the prototype MPAC and acts by competing with proteins for metal ions [100]. Clioquinol (CQ) is an orally bioavailable drug with moderate affinity for copper, zinc and iron. Differing from traditional chelators as mentioned above, these compounds do not remove metals from tissues. CQ appears to act as an ionophore to redistribute metals from areas of superabundance to those which may be deficient. Unlike traditional chelators such as EDTA, CQ does not cause bulk excretion of metals but permeates the BBB and potently inhibits metal-mediated hydrogen peroxide production [18]. CQ and analogues are being investigated in a number of conditions in which oxidative stress is a feature. These include; cancer [97, 98] stroke [272], AD [336, 343] and PD [207].

This type of therapeutic approach using CQ type MPACs appears to be encouraging for AD. Animal trials with 21 month old transgenic mice over expressing the amyloid precursor protein (APP) with the Swedish mutation showed a significant reduction in Aβ plaques after treatment with CQ [60]. A phase II double-blinded clinical trial showed that CQ treatment for 36 weeks resulted in a reduction of Aβ1-42 in plasma, with minimal cognitive decline [343]. While this study had a very small subset which reflected within the non significant difference between the groups, these results support the idea that metals play an important role in neurological diseases.

There is also proof of concept that MPACs may be useful for PD therapeutics. A parkinsonian animal model study showed that treatment of animals with CQ for eight weeks prior to induction of lesions resulted in 50% decrease in nigral cell loss compared to animals treated with the parkinsonian toxin MPTP alone [207]. An 8 week pretreatment of CQ resulted in reduction in iron within the SN in MPTP lesioned mice [146]. More recently data showing that CQ treatment commencing only 6 hours after induction of the lesion is equally effective at attenuating SN lesions provoked by intra-nigral injection of 6-OHDA [428] Further the data from a neuronal cell line that expresses the A30P mutant human α-synuclein was rescued by either catalase or CQ [428].

## CONCLUSION

Understanding the causes and the pathology of PD is pivotal in identifying specific targets for drug intervention. Surgical interventions have a limited niche in the treatment of symptoms of PD, while other restorative measures (such as gene therapy) are still experimental and pre-clinical. The challenge is to develop novel therapeutic agents which are capable of slowing SN cell loss and reducing disease progression. Recent studies into new classes of drugs (such as MPACs) have shown that they may intervene with interactions between dopamine, alpha synuclein and redox-active metals. This class of therapeutic drug offers a new pharmacological approach that could potentially modify the progression of PD.

## Figures and Tables

**Table 1:** Genes and their Associated Mutations that have been Identified in PD

Gene	Function	Major Mutations Identified	Mode of Inheritance	Clinical	Pathology (Human)	Pathology *In Vivo* Model
*α-Syn [198, 333](PARK1)*	Possibly functions as a neurotransmitter modulator	A53T, A30P, E46K	Autosomal dominant	Similar to sporadic PD, early onset	SN depigmentation, DA loss, gliosis, LB & neuritis [382] Increases in amyloidal fibrillation and tau inclusion [156]LBs	DA loss, LB are present in areas which are not associated with PD [204, 227]
*Parkin(PARK2)*	E3 Ubiquitin protein ligase [361]	EX3-7DEL EX4DEL T240N [169, 170, 215, 256, 273]	Autosomal recessive	Early onset, slow progression parkinsonism [361]	Variations in the presence of LB	Data not available
*(PARK3)*	Unknown	Data not available	Autosomal dominant	Data not available	Data not available	Data not available
*(PARK4)*	See *α-Syn* above	*α-Syn * Triplication (up to 4 full copies)	Autosomal dominant	Early Onset PD	Nigral and locus coeruleus degeneration, presence of LB in hippocampus, locus coeruleus and cortices [297, 368]	Data not available
UCH L1 (PARK5)	C-terminal ubiquitin hydrolyse and, ubiquitin ligase [241, 250]	V66M, S18Y, I93M [216, 262, 290, 351, 393, 401, 430, 444]	Autosomal dominant	PD	Data not available	Mutant mice display gracile axonal dystrophy [213]
*PINK1(PARK6)*	Mitochondrial serine/threonine kinase [230, 231]	H271Q, L347P, 1573TTAG, 1602CAA, R279H, DEL EX6-8, T313M, A217N L489P, L347P, E240K, A340T [132, 402, 420]	Autosomal recessive	Similar to atypical sporadic PD, early onset	Data not available	PINK1 silencing showed rapid eye degeneration and progressive DA loss in a drosophila model [419, 435]
*DJ-1(PARK7)*	Protection against oxidative stress [196, 286, 288]	14-KB DEL, L166P, M26I, D149A, G64D, E163K + 18-BP DUP	Autosomal recessive	Early onset PD	Data not available	DJ-1 null mice showed no loss of striatal DA neurons [213]
*LRRK2(PARK8)*	Protein kinase (unknown substrates)	R1441G, Y1699C, R1441C, L1122V, G2019S, I2020T, R1441H, G2385R, P755L, [85]	Autosomal dominant	PD	LB, nigral degeneration without LB, And tau aggregation [452]	Data not available
